# Screen time and physical activity in children and adolescents aged 10–15 years

**DOI:** 10.1371/journal.pone.0254255

**Published:** 2021-07-09

**Authors:** Anna Dahlgren, Linnea Sjöblom, Helén Eke, Stephanie E. Bonn, Ylva Trolle Lagerros

**Affiliations:** 1 Clinical Epidemiology Division, Department of Medicine (Solna), Karolinska Institutet, Stockholm, Sweden; 2 Center for Obesity, Academic Specialist Center, Stockholm Health Services, Stockholm, Sweden; University of New Brunswick, CANADA

## Abstract

**Background:**

Previous research studies have demonstrated a relationship between low levels of physical activity and high amounts of screen time in children and adolescents. However, this is usually based on self-reported data. Therefore, the aim of this cross-sectional study was to investigate the association between objectively measured smartphone screen time and physical activity among children and adolescents aged 10–15 years.

**Methods:**

During seven consecutive days, we objectively assessed smartphone screen time, using the SCRIIN smartphone application, and physical activity, using the SCRIIN activity tracker, in children and adolescents recruited from two schools in Stockholm County, Sweden. Moreover, the children/adolescents and their parents responded to a questionnaire, obtaining among other things: self-reported screen time, physical activity, sleep and health-related quality of life.

**Results:**

A total of 121 children and adolescents (mean age: 12.1 ± 1.5) were included in the study. Objectively measured smartphone screen time was 161.2 ± 81.1 min/day. Mean physical activity, measured with the SCRIIN activity tracker, was 32.6 ± 16.5 active min/day. Minutes of screen time and physical activity did not differ between the children and adolescents from the two schools, despite located in different socioeconomic areas. Further, we found no association between smartphone screen time and physical activity. However, girls aged 14–15 years, had more smartphone screen time (p<0.01) and were significantly more physically active (p<0.01) than girls aged 10–12 years. In addition, boys reported more than five times more time spent on computer and video games than girls did.

**Conclusion:**

Smartphone screen time was not associated with physical activity level among children and adolescents aged 10–15 years.

## Introduction

Regular physical activity reduces the risk of childhood and adolescence overweight and obesity, and future associated chronic diseases [1]. However, results from a recent report indicated that children and adolescents in Sweden spend most of their time being inactive. Among 11 year-olds, only 14% of the girls and 23% of the boys reached the daily recommendation of at least 60 minutes of moderate-to-vigorous physical activity (MVPA). Additionally, the time spent being physically active seems to decrease with age. Only 9–15% of adolescents aged 13–15 years reached the recommendation [2]. At the same time, the amount of screen time in this population is high: 30% of 11-year-olds reported a screen time of more than 4 hours per day, while the corresponding number for 15-year-olds was 51% [3]. Generally, screen time was higher in boys than in girls.

High amounts of screen-based sedentary behaviours, along with insufficient physical activity, are associated with a large range of physical and psychological disorders, which may affect health and wellbeing negatively [4]. These behaviours are considered increasing public health concerns, especially in children, as prolonged screen time and low levels of physical activity are suggested as unhealthy behaviours that may persist into adulthood [5]. Additionally, previous studies have reported significant associations between more screen time and lower levels of physical activity [6,7] in children and adolescents, indicating that sedentary screen time is likely spent at the expense of other healthy activities.

In order to measure physical activity and screen time, reliable assessments are needed. Both accelerometers and questionnaires have been widely used to measure level of physical activity. However, questionnaires commonly overestimate active time and underestimate sedentary behaviours [8]. Methods for measuring screen time are limited, and studies using objective measures capturing real-time use of screen devices are scarce. A previous study among university students, objectively tracked daily minutes of screen time using a smartphone application [9]. Similarly, a cross-sectional study in adults also measured smartphone screen time continuously via an application [10]. Nevertheless, as far as we know, only questionnaires have been used to assess amount of screen time among children and adolescents.

The extensive use of screen devices has led to an increased interest in using new technologies such as smartphone applications in research studies, for example to investigate different behaviours. Therefore, in this study, we evaluated the association between smartphone screen time, objectively measured with a smartphone application, and objectively measured physical activity among children and adolescents aged 10–15 years. Our hypothesis was that children and adolescents with lower smartphone screen time would be more physically active.

## Materials and methods

### Study design

The Swedish SCRIIN-study is a school based cross-sectional study. The recruitment to the study took place in April 2019 in two schools in the Stockholm County of Sweden. The study was approved by the Swedish Ethical Review Authority (Dnr: 2019–01336) and the research was conducted at Karolinska Institutet.

### Inclusion criteria

Inclusion criteria were: being a pupil in fifth (10–12 years) or eighth (14–15 years) grade in one of the two participating schools, having access to a smartphone, giving informed consent and having parents or caretakers both giving informed consent for their child/adolescent to participate in the study and, if in fifth grade, to download the Swedish SCRIIN smartphone application.

### Exclusion criteria

No specific exclusion criteria applied.

### Recruitment

Information about the study was disseminated via the Swedish Facebook group “Physical activity for Health and Learning”. Teachers interested to take part with their classes were encouraged to get in touch through email. We were contacted by several teachers, but chose to collaborate with two physical education teachers from two schools situated in socioeconomically different areas of Stockholm. Demographic details of the municipalities for the schools included in this study can be found in supporting information, S1 Table.

We invited the children and adolescents from their ten classes, four fifth grade and six eighth grade classes, to participate in the study. Parents of children and adolescents in fifth and eighth grade received oral and written information about the study, both through a meeting in the school and through the ordinary weekly newsletters from the school. An email including a link to a web-questionnaire was sent to the parents. After an introductory screen displaying information about the study, parents’ consented to participate together with their child or adolescent, either electronically or in writing, and were thereafter offered to respond to the parental questionnaire (see supporting information, S2 Table). Once both parents had provided written informed consent, the child/adolescent was eligible to enter the study. The children and adolescents got oral and written information about the study in school. They too gave their written informed consent after reading information about the study, before responding to their web-questionnaire (see supporting information, S3 Table). Children/adolescents and parents were informed that participation in the study was voluntary and that they could withdraw their consent at any time.

### Study assessments

Participant characteristics were collected from the children/adolescents and parents with questions about their age and gender, and parents were in addition asked about their level of education. The Kid- and Kiddo-KINDL–health-related quality of life questionnaire was used to assess how the child/adolescent perceived physical- and emotional well-being, self-esteem, family life, friends and everyday functioning in school [11]. The validated Sedentary Behaviour Questionnaire (SBQ) was used to collect data about screen time and sedentary time among both children/adolescents and parents [12]. Physical activity level, body weight and height were also included in both questionnaires. Body weight and height were used to calculate Body Mass Index (BMI, kg/m^2^). When classifying BMI in the children/adolescents, the international classification system for childhood obesity (isoBMI) recommended by the International Obesity Task Force was used [13].

The SCRIIN smartphone application automatically recorded screen time. Screen time was defined as any time when the smartphone screen was on and unlocked. Therefore, the children and adolescents were asked to ensure that the automatic screen lock was activated and set to 30 seconds to make the recorded data as accurate as possible.

The children and adolescents wore the SCRIIN activity tracker on the hip during waking hours for seven consecutive days. The tracker used an accelerometer-based technology that converted acceleration into steps and active minutes. An active minute was recorded when MVPA was reached throughout the minute, which was defined as at least 80 steps per minute. Minutes that were not classified as active were defined as light activity/inactive time.

In our initial validation tests of the SCRIIN activity tracker, we found that the commonly used 100 steps per minute threshold underestimated MVPA. Therefore, we chose a lower threshold, 80 steps per minute. Since this was an arbitrary number, we decided to conduct a validation study of the SCRIIN activity tracker against an accelerometer, the ActiGraph wGT3x-BT.

The information about smartphone screen time, active and light activity/inactive time was not displayed in the application during the time of measurement. Furthermore, as the SCRIIN activity tracker stored the physical activity data and automatically connected to the application via Bluetooth when the smartphone was nearby, the smartphone did not have to be carried around at all times. A minimum of three days using the SCRIIN application and/or SCRIIN activity tracker was required to be included in the data analysis.

The SCRIIN activity tracker is publicly available (www.sportamore.com). The SCRIIN smartphone application can be downloaded via AppStore or Google Play and is compatible with both iOS (version 8 and higher) and Android (version 4.1 and higher).

### Validation of the Swedish SCRIIN activity tracker against ActiGraph wGT3x-BT

In order to validate the SCRIIN activity tracker, the triaxial accelerometer ActiGraph wGT3x-BT (www.actigraphcorp.com), was given to a sub-sample of children in fifth grade. Participants carried both the accelerometer and the SCRIIN activity tracker for 5.5 hours (330 minutes) during a school day, either between 8:00 am to 1:30 pm or 9:00 am to 2:30 pm. The accelerometer was worn on the hip, collecting data at the frequency of 80 Hz. The validation included 35 participants, whom had data from both the accelerometer and the SCRIIN activity tracker. Analysis of data was performed in ActiLife, version 6.13.3, where the three axes were calculated to a vector magnitude and summarized as counts per minute (cpm). The cut-point for MVPA was applied as suggested by Hänggi et al. [14], which is recommended for children/adolescents in this age group in the review by Migueles et al. [15]. An epoch length of 60 seconds and normal filter was used. Non-wear time was defined as 20 consecutive minutes with 0 cpm [16].

### Statistical analysis of data

In total, data from 121 children/adolescents and 88 parents were included in the analyses. Descriptive statistics were summarized to describe participant characteristics, and is presented as mean and standard deviation (SD) for continuous variables and as number (n) and percentage (%) for categorical variables. Differences in characteristics when stratified by gender and age, were examined using independent t-tests and chi-square tests for continuous and categorical variables, respectively. Spearman’s rank correlation tests were conducted for evaluating the association between physical activity and screen time among children and adolescents. Objectively measured smartphone screen time from the SCRIIN application and self-reported smartphone/tablet screen time from the questionnaire was compared using a paired t-test and Bland-Altman analysis. When validating the SCRIIN activity tracker against the accelerometer, a paired t-test, Spearman’s rank correlation test, and Bland-Altman analysis were used. All reported *p*-values were two-sided and *p*-values <0.05 were considered statistically significant. The statistical analyses of data were performed in STATA, version 15.1.

## Results

Study characteristics of all children and adolescents included in the study are presented in Table 1. The average isoBMI was 19.4 ± 3.9 kg/m^2^. The distribution in isoBMI categories differed between the two schools. The proportion of overweight and obesity was significantly higher in the school located in the area with low socioeconomic status, where the rate of overweight and obesity was 31.0% compared to 6.0% in the school with high socioeconomic status.

**Table 1:** Characteristics of the children and adolescents, all and stratified by grade and sex.

		Fifth grade		Eighth grade	
	All	Boys	Girls	Boys	Girls
	(n = 121)	(n = 40)	(n = 38)	(n = 27)	(n = 16)
	**Mean** ± **SD**	**Mean** ± **SD**	**Mean** ± **SD**	**Mean** ± **SD**	**Mean** ± **SD**
Age (years)	12.1 ± 1.5	11.0 ± 0.2	11.0 ± 0.2	14.0 ± 0.0	14.1 ± 0.3
isoBMI (kg/m^2^)	19.4 ± 3.9^a^	18.6 ± 3.8^b^	19.2 ± 4.3^c^	19.7 ± 3.6^d^	21.6 ± 3.0^e^
**isoBMI category**	**n (%)**	**n (%)**	**n (%)**	**n (%)**	**n (%)**
Normal weight	76 (82.6)	25 (83.3)	22 (81.5)	19 (82.6)	10 (83.3)
Overweight	10 (10.9)	3 (10.0)	2 (7.4)	3 (13.0)	2 (16.7)
Obese	6 (6.5)	2 (6.7)	3 (11.1)	1 (4.4)	0 (0.0)
**Kid- and Kiddo KINDL HRQoL**	**Mean** ± **SD**	**Mean** ± **SD**	**Mean** ± **SD**	**Mean** ± **SD**	**Mean** ± **SD**
Physical well-being	31.3 ± 10.9	30.9 ± 11.2	33.8 ± 11.5	26.7 ± 9.3	33.6 ± 9.4
Emotional well-being	32.6 ± 8.6	32.7 ± 7.5	34.5 ± 8.4	30.0 ± 10.3	32.0 ± 8.5
Self-esteem	62.2 ± 23.5	57.1 ± 24.3	59.5 ± 21.0	70.9 ± 24.0	66.4 ± 23.4
Family	47.5 ± 9.4	45.6 ± 11.9	47.1 ± 7.9	48.6 ± 7.4	51.2 ± 8.0
Friends	60.9 ± 14.2	56.3 ± 18.2	62.5 ± 11.1	63.7 ± 10.8	63.3 ± 13.5
Everyday functioning in school	42.9 ±13.2	37.3 ± 14.8	44.4 ± 9.5	48.1 ± 13.1	44.1 ± 13.2
**Sleep**					
Wake up time (hh:mm)	06:36 ± 00:28	06:38 ± 00:31	06:27 ± 00:26	06:46 ± 00:28	06:34 ± 00:19
Bed time (hh:mm)	21:54 ± 00:47	21:43 ± 00:46	21:28 ± 00:32	22:25 ± 00:43	22:26 ± 00:41
Sleep time (h)	8.7 ± 0.81	8.9 ± 0.9	9.0 ± 0.6	8.3 ± 0.6	8.1 ± 0.8

BMI = Body Mass Index, HRQoL = Health-related quality of life.

^a^n = 92,

^b^n = 30,

^c^n = 19,

^d^n = 23,

^e^n = 12.

In Table 2, parental characteristics are presented. The mean age of the parents was 45.4 ± 5.7 years and the mean BMI was 25.4 ± 3.6 kg/m^2^. In the school situated in the area with low socioeconomic status, the percentage of overweight and obesity was also higher among the parents. The frequency of overweight and obesity in parents was 45.9% and 22.6%, respectively. Additionally, the level of education was significantly lower among parents in the area with low socioeconomic status, where 15.0% had elementary school as the highest completed education. The proportion of individuals born outside the Nordic countries or outside Europe was also higher (45.0%) among these parents, while in the school located in the area with high socioeconomic status, only 4.4% of the parents were born outside the Nordic countries.

**Table 2:** Characteristics of parents.

	All	Women	Men
	(n = 88)	(n = 53)	(n = 35)
	**Mean** ± **SD**	**Mean** ± **SD**	**Mean** ± **SD**
Age (years)	45.4 ± 5.7	43.6 ± 5.0	47.8 ± 5.7
BMI (kg/m^2^)	25.4 ± 3.6	25.1 ± 4.2	26.0 ± 2.3
**Education**	**n (%)**	**n (%)**	**n (%)**
Elementary school	6 (6.8)	6 (11.3)	0 (0.0)
2 year high school	10 (11.4)	6 (11.3)	4 (11.4)
3 year high school	31 (35.2)	22 (41.5)	9 (25.7)
University/college	41 (46.6)	19 (35.9)	22 (62.9)
**Country of birth**			
Sweden or other Nordic country	66 (75.0)	38 (71.7)	28 (80.0)
Other European country	7 (8.0)	5 (9.4)	2 (5.7)
Other country outside Europe	15 (17.1)	10 (18.9)	5 (14.3)
**Screen time**	**Mean** ± **SD**	**Mean** ± **SD**	**Mean** ± **SD**
Smartphone/tablet (min/day)	96.7 ± 59.9	108.9 ± 60.4	78.5 ± 55.1
TV (min/day)	81.3 ± 55.8	87.8 ± 60.7	71.7 ± 46.9
Computer/TV games (min/day)	2.0 ± 10.3	2.8 ± 12.7	0.9 ± 5.1
Screen time total (min/day)	127.5 ± 68.0	142.4 ± 71.3	105.5 ± 57.0
**Physical activity–daily occupation**	**n (%)**	**n (%)**	**n (%)**
1 *Mostly sitting*	25 (28.4)	14 (27.0)	11 (31.4)
2	19 (21.8)	7 (13.5)	12 (34.3)
3 *Mostly standing/walking*	31 (35.6)	25 (48.1)	6 (17.1)
4	7 (8.1)	6 (11.4)	1 (2.9)
5 *Heavy labour*	5 (5.8)	0 (0.0)	5 (14.3)
**Physical activity–leisure time**			
1 *Mostly sitting*	6 (7.1)	5 (10.2)	1 (2.9)
2	11 (13.1)	8 (16.3)	3 (8.6)
3 *Walking 30 min/day*	28 (33.3)	18 (36.7)	10 (28.6)
4	26 (31.0)	11 (22.5)	15 (42.9)
5 *Vigorous activity 60 min or more/day*	13 (15.5)	7 (14.3)	6 (17.1)

BMI = Body Mass Index.

### Screen time

The average objectively measured smartphone screen time was 161.3 ± 82.0 min/day, presented in Table 3. The amount of screen time did not differ between the schools. Mean daily smartphone screen time was 163.8 ± 80.9 min for girls and 156.1 ± 84.6 min for boys.

**Table 3:** Screen time from SCRIIN application and questionnaire, all and stratified by grade and sex.

	All	Fifth grade			Eighth grade		
		Boys	Girls		Boys	Girls	
	(n = 83)	(n = 18)	(n = 31)		(n = 20)	(n = 9)	
**SCRIIN application**	**Mean** ± **SD**	**Mean** ± **SD**	**Mean** ± **SD**	*p*-value	**Mean** ± **SD**	**Mean** ± **SD**	*p*-value
Smartphone (min/day)	161.3 ± 82.0	164.8 ± 66.1	146.3 ± 76.0	0.39	148.3 ± 99.4	224.3 ± 70.2	0.08
	(n = 117)	(n = 38)	(n = 37)		(n = 26)	(n = 16)	
**Questionnaire**	**Mean** ± **SD**	**Mean** ± **SD**	**Mean** ± **SD**		**Mean** ± **SD**	**Mean** ± **SD**	
Smartphone/tablet (min/day)	149.6 ± 103.1	130.1 ± 90.7	144.0 ± 101.1	0.53	134.6 ± 102.4	232.9 ± 105.2	<0.01
TV (min/day)	111.0 ± 79.6	110.5 ± 92.1	96.0 ± 70.8	0.45	128.8 ± 72.4	118.4 ± 78.4	0.68
Computer/video games (min/day)	93.7 ± 113.7	165.3 ± 126.4	31.8 ± 63.7	<0.0001	123.4 ± 106.7	18.5 ± 43.3	<0.001
Screen time total (min/day)	354 ± 198.9	406.0 ± 235.9	271.8 ± 177.2	<0.01	386.4 ± 170.6	369.8 ± 138.7	0.75
**Opinions about screen time**	**n (%)**	**n (%)**	**n (%)**		**n (%)**	**n (%)**	
Want to reduce my screen time	48 (44.0)	9 (25.7)	21 (58.3)	<0.01	9 (37.5)	9 (64.3)	0.24
Satisfied with my screen time	55 (50.5)	21 (60.0)	15 (48.7)		14 (58.3)	5 (35.7)	
Want more screen time	6 (5.5)	5 (14.3)	0 (0.0)		1 (4.2)	0 (0.0)	

When stratifying by gender, objectively measured smartphone screen time was significantly higher among girls in eighth grade than girls in fifth grade (p<0.01). Girls in eighth grade, also had significantly higher self-reported smartphone/tablet screen time and total screen time, compared to girls in fifth grade. Average self-reported smartphone/tablet screen time for girls, in all ages, was 170.8 ± 109.4 min/day, while total screen time was 301.4 ± 171.3 min/day. The girls reported that they were satisfied, or wanted to reduce their screen time. No girl wanted to increase her screen time.

The mean self-reported smartphone/tablet screen time for boys in all ages was 132.0 ± 94.9 min/day and total screen time was 398.0 ± 210.5 min/day. No statistical difference was shown between boys in different ages regarding screen time. However, the amount of time spent on computer and video games differed significantly between genders, where boys reported 148.3 ± 119.7 min/day and girls 27.8 ± 58.2 min/day. In contrast to the girls, boys reported that they wanted more screen time.

Among parents, mean self-reported smartphone/tablet screen time was 96.7 ± 59.9 min/day and total screen time was 127.5 ± 68.0 min/day. Both smartphone/tablet screen time and total screen time differed significantly between the genders. For example, women reported 108.9 ± 60.4 min/day of smartphone/tablet screen time, while men reported 78.5 ± 55.1 min/day. The amount of parental screen time was not correlated with the amount of screen time in children and adolescents (r = 0.04, p = 0.70).

### Objectively measured vs self-reported smartphone screen time

**Fig 1:**
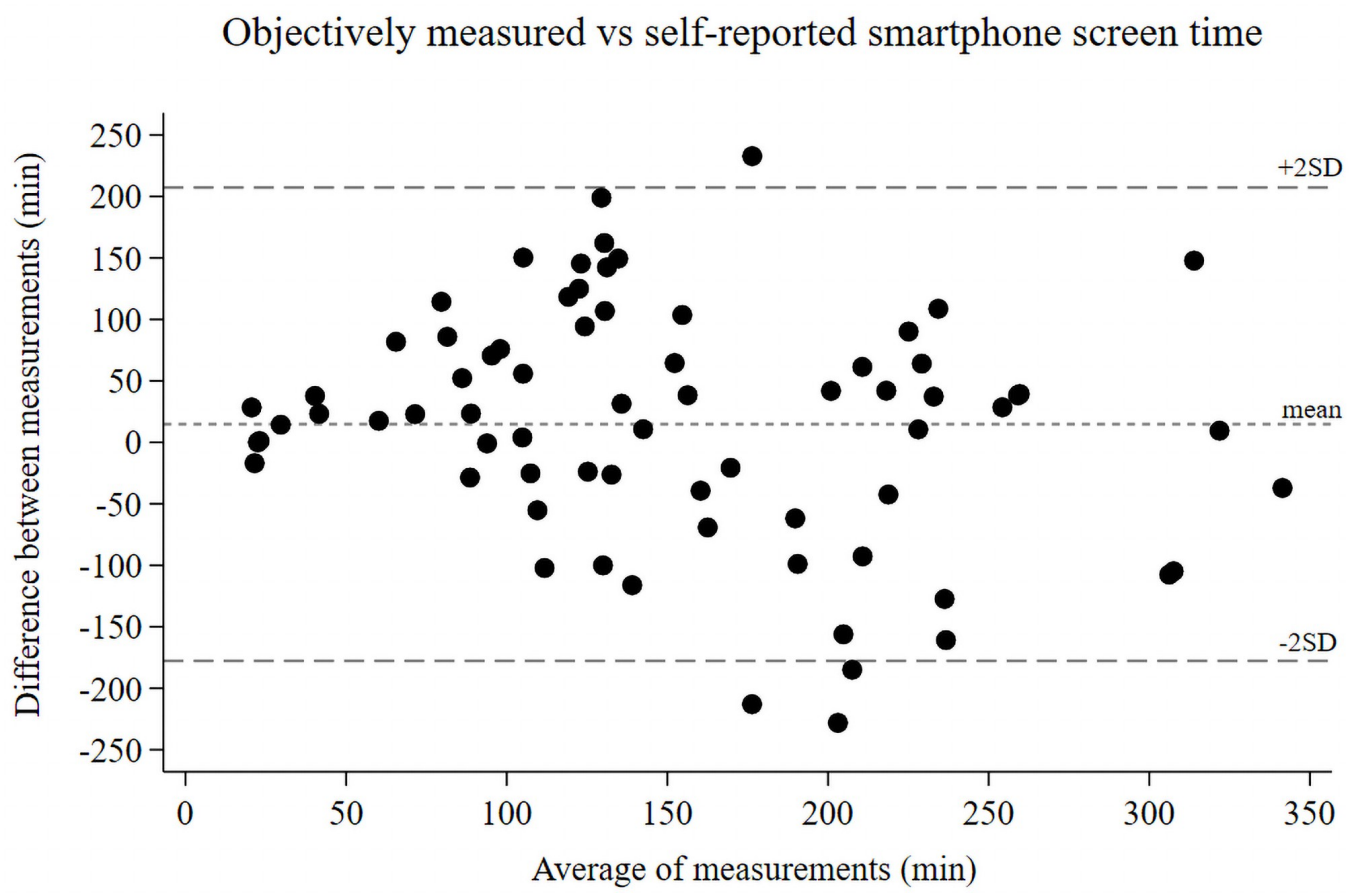
Bland-Altman plot. Comparing smartphone screen time measured with SCRIIN application and smartphone/tablet screen time from questionnaire.

A Bland-Altman plot visualizes the comparison between objectively measured smartphone screen time with SCRIIN application and self-reported smartphone/tablet screen time from questionnaire (Fig 1). Even though the measurement methods do not differ significantly on group level, there are large variations on individual level.

### Physical activity

As outlined in Table 4, 77 children and adolescents had data from the SCRIIN activity tracker, and 119 children and adolescents had self-reported questionnaire data. Average physical activity, measured with SCRIIN activity tracker, was 32.6 ± 16.5 active min/day. Mean physical activity time was 33.2 ± 14.1 active min/day for girls and 31.9 ± 18.6 active min/day for boys. The amount of physical activity was significantly higher in girls in eighth grade, compared to girls in fifth grade (p<0.01). No difference was found regarding minutes of physical activity between the schools.

**Table 4:** Physical activity from SCRIIN activity tracker and questionnaire, all and stratified by grade and sex.

		Fifth grade			Eighth grade		
	All	Boys	Girls		Boys	Girls	
	(n = 77)	(n = 18)	(n = 28)		(n = 18)	(n = 8)	
**SCRIIN activity tracker**	**Mean** ± **SD**	**Mean** ± **SD**	**Mean** ± **SD**	*p*-value	**Mean** ± **SD**	**Mean** ± **SD**	*p*-value
Physical activity (active min/day)	32.6 ± 16.5	35.1 ± 18.5	30.0 ± 10.8	0.24	28.7 ± 18.7	44.5 ± 18.9	0.06
	(n = 119)	(n = 39)	(n = 37)		(n = 27)	(n = 16)	
**Questionnaire**	**Mean** ± **SD**	**Mean** ± **SD**	**Mean** ± **SD**		**Mean** ± **SD**	**Mean** ± **SD**	
School sport lesson (min/day)	25.6 ± 4.1	26.5 ± 4.9	24.3 ± 2.0	0.02	26.7 ± 5.0	24.8 ± 3.0	0.18
Physical activity evening (min/day)	80.3 ± 54.5	60.4 ± 47.9	108.9 ± 50.5	<0.001	84.6 ± 55.6	57.0 ± 50.5	0.12
Physical activity weekend (min/day)	84.6 ± 58.7	78.2 ± 57.3	111.6 ± 56.3	0.02	73.8 ± 53.1	58.0 ± 58.3	0.38
Physical activity total (min/day)	101.6 ± 49.7	85.7 ± 45.2	129.5 ± 44.8	<0.001	104.3 ± 48.3	75.0 ± 46.2	0.07
**Opinions about physical activity**	**n (%)**	**n (%)**	**n (%)**		**n (%)**	**n (%)**	
Want more physical activity	74 (67.3)	24 (64.9)	24 (70.6)	0.76	13 (56.5)	13 (81.3)	0.07
Satisfied with my physical activity level	36 (32.7)	13 (35.1)	10 (29.4)		10 (43.5)	3 (18.8)	

According to self-reported data, mean total physical activity of MVPA was 112.5 ± 51.3 min/day for girls and 93.0 ± 46.9 min/day for boys. Total daily physical activity and physical activity levels during evenings and weekends were significantly higher among girls in fifth grade, compared to boys of the same age. A high proportion of the children and adolescents responded that they wanted to be more physically active, as many as 74.0% of the girls and 61.7% of the boys.

### Screen time vs physical activity

No correlation was observed between objectively measured smartphone screen time and objectively measured physical activity for the entire study population of children and adolescents (r = 0.15, p = 0.21), nor when stratified by gender and/or age. Likewise, no correlation was shown between self-reported smartphone screen time and objectively measured physical activity for the total study population (r = 0.04, p = 0.78), or for girls in fifth grade (r = -0.26, p = 0.19) or girls in eighth grade (r = -0.65, p = 0.08). However, among boys in fifth grade, a weak correlation was observed between self-reported smartphone screen time and objectively measured physical activity (r = 0.51, p = 0.03). No such correlation was shown for boys in eighth grade (r = 0.04, p = 0.88).

### Validation of SCRIIN activity tracker against ActiGraph wGT3x-BT

**Fig 2:**
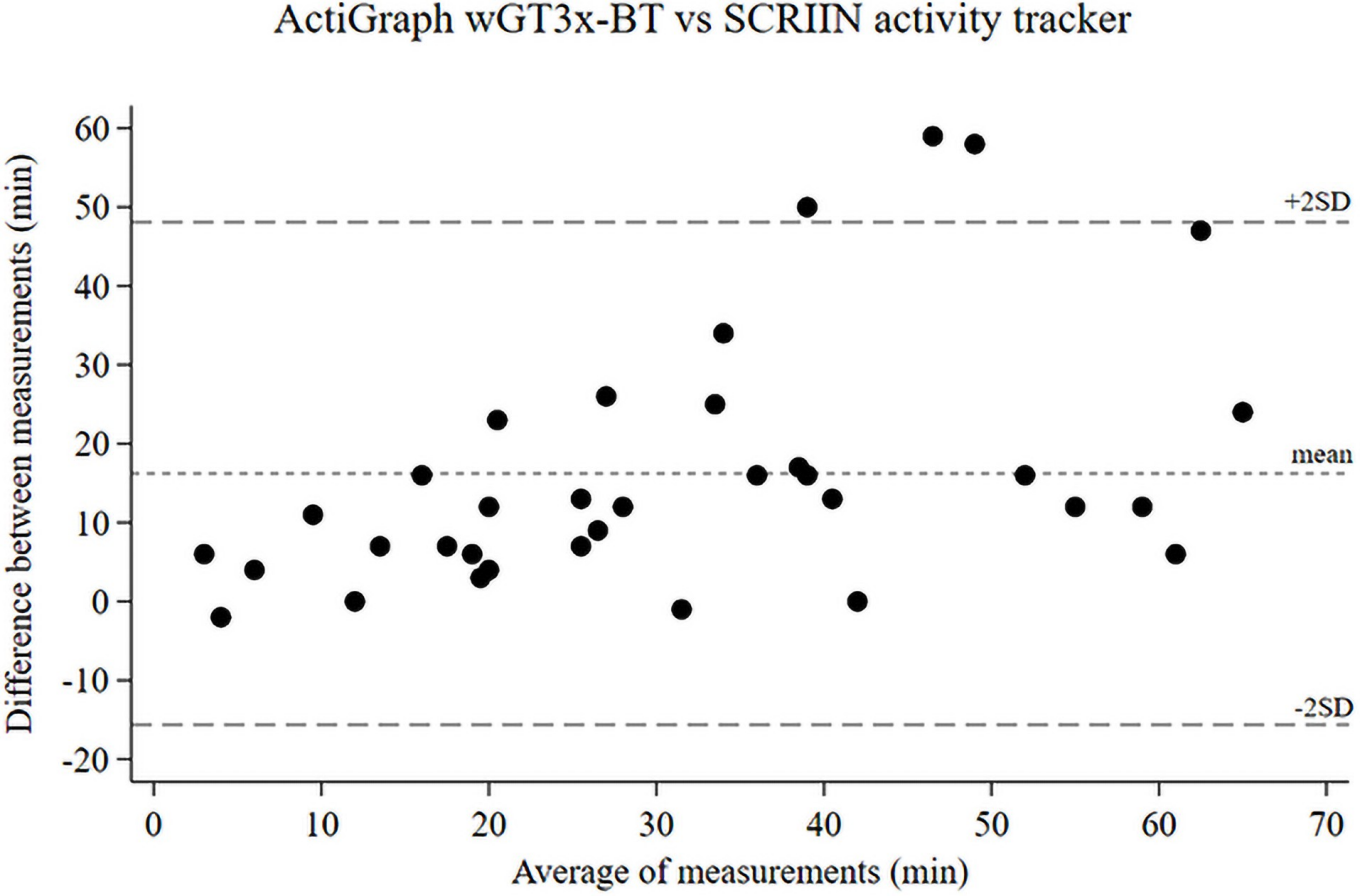
Bland-Altman plot. Comparing physical activity measured with ActiGraph wGT3x-BT in moderate-to-vigorous physical activity and the Swedish SCRIIN activity tracker in active minutes.

The SCRIIN activity tracker measured on average 23.2 ± 15.3 active minutes compared to the accelerometer ActiGraph wGT3x-BT, which on average measured 39.5 ± 22.3 minutes of MVPA. Results from Spearman’s rank correlation demonstrated a significant correlation (r = 0.72, p<0.001) between active minutes measured by the SCRIIN activity trackers and minutes of MVPA measured by the accelerometers. Thus, the SCRIIN activity tracker and the accelerometer assessed the participants similarly in terms of physical activity level. However, the absolute difference between the measurement tools was 16.2 minutes (p<0.001). The Bland-Altman plot (Fig 2) shows wide limits of agreement between the ActiGraph wGT3x-BT and the SCRIIN activity tracker. The outliers are placed above the limits of agreement with a physical activity level over 40 minutes, i.e. the SCRIIN activity tracker tended to underestimate physical activity compared to the accelerometer, especially when the level of activity was high.

## Discussion

In this cohort of children and adolescents from two municipalities in Sweden, we found no association between objectively measured smartphone screen time and objectively measured physical activity, i.e. the time spent using their smartphone was not associated with the time spent being physically active, in any of the age or gender groups. Girls aged 14–15 years, however, had both significantly more smartphone screen time and higher physical activity level, compared to girls aged 10–12 years. Further, boys reported more than five times more time of playing video and computer games than girls did. No association between parents´ and children’s/adolescents´ amount of screen time was found.

Similar to our study, Auhuber et al. found no association between smartphone screen time and total physical activity in children and adolescents of similar age [17]. The differences they found between boys and girls were also consistent with the results of our study, where girls reported a higher smartphone screen time, and boys spent more time playing computer or video games. However, evidence from earlier studies demonstrates an inverse relationship between physical activity and screen time [6,7]. For example, Sandercock et al. found a significant negative association between screen time and physical activity in children and adolescents aged 10–16 years [6]. Additionally, a study conducted among children, aged 7–12 years, demonstrated a relationship between low physical activity level and high self-reported screen time [7]. Another study indicated a modest, inverse association between screen time and MVPA among children aged 9–15 years [18].

Differences in approaches for measuring screen time make comparison with earlier research difficult. While previous studies exclusively have used questionnaires to measure screen time, this study is, to our knowledge, the first to measure smartphone screen time objectively in children and adolescents. Our results demonstrated large individual differences between objectively measured and self-reported screen time, which indicates uncertainties about self-reported data. However, it should be noted that we analysed smartphone screen time separately, which differs from the majority of previous research studies where screen time was not divided into different subcategories [6,7,18]. Thereby it is difficult to determine which screen device that was used.

In contrast to previous research, we observed that girls aged 14–15 years had a higher objectively measured physical activity level than girls aged 10–12 years. Girls aged 14–15 years also seemed to be more physically active than both the younger boys and those in the same age group, which likewise is inconsistent with earlier findings. It should be noted though, that our sample of eighth grade girls is small. Girls 10–12 years, however, self-reported more physical activity than both the boys in the same age and the older girls did.

Guthold et al., investigated the global prevalence of insufficient physical activity among children and adolescents aged 11–17 and found, on the contrary, girls to be more physically inactive than boys [19]. Moreover, Rosselli et al. assessed gender differences in physical activity and discovered that girls less frequently reached the recommended level of physical activity [20]. In another study, where patterns and determinants of MVPA among youths were analysed, results showed that boys were more active than girls were by the age of 15 years Additionally, physical activity decreased significantly with age [21]. This is consistent with another study conducted by Trost et al., where a significant inverse relationship was found between grade level and daily MVPA [22]. It is unclear to us why the girls in our study were more physically active than the boys. Although, not confirmed in our study, it is more common that Swedish boys are overweight and obese than the girls, and the risk of obesity is increasing with age, especially in boys [23].

Our study includes various strengths and limitations that should be taken into account. The ability to measure smartphone screen time objectively among children and adolescents is a strength. The SCRIIN application provides detailed information on smartphone screen time over the period of measurement. Although Swedish statistics from 2019 show that daily internet connection most commonly is done from a smartphone [24], future studies should consider assessing the amount of screen time from other devices as well. Another strength is the high correlation between the SCRIIN activity tracker and the ActiGraph accelerometer in the validation we conducted as a part of this study. The accelerometer ActiGraph wGT3x-BT is widely used to capture and record continuous, high-resolution physical activity data in different study populations [15]. The accelerometer cut-point we used was developed for children, and has shown good agreement when validated [14].

The fact that we included schoolchildren and adolescents from both fifth and eighth grade is an advantage in this study. This made it possible to identify age-related differences. To increase generalizability, the recruitment of children and adolescents took place in schools located in two socioeconomically different areas; our sample can be considered representative of a larger population. In the municipality with low socioeconomic status, the parents were less educated and more commonly born outside of Sweden. We also observed a higher proportion of overweight and obesity in both children/adolescents and parents in the municipality with low socioeconomic status, compared to the municipality with high socioeconomic status. The latter is consistent with statistics from the Swedish Public Health Authority, reporting the percentage of people with obesity to be 20% respectively 13% for the two municipalities where the study was conducted [25]. Despite these demographic differences, there was no difference in either screen time or physical activity between the children/adolescents in the different schools.

Among the limitations is the possibility of both under- and overreporting of self-reported data. This could for instance arise from social desirability [26]. For example, none of the participating girls in our study wanted more screen time. This warrants more research, as we cannot be sure if this is due to social desirability or other reasons. Another limitation is the missing or incompleteness of data from the SCRIIN application and the SCRIIN activity tracker for some of the children and adolescents. In order to download the application, they needed to have access to a smartphone, and in addition, for fifth grade, have parents giving informed consent for downloading the application. Thus, for some children and adolescents, we only have questionnaire data. The use of the activity tracker largely depended on memory and motivation of the carrier. Some children and adolescents forgot to put the activity tracker on, or inadvertently left it on another piece of clothing. For this reason, we had a minimum of three measured days as a criterion to be included in the analysis.

A commonly used cut-point for MVPA is 100 steps per minute [27–29]. We used 80 steps per minute as cut-point, which could have resulted in overestimation of active minutes. Nevertheless, our validation of the SCRIIN activity tracker against the ActiGraph wGT3x-BT showed the contrary, i.e. the SCRIIN activity tracker underestimated MVPA. Consequently, an even lower cut-point for the SCRIIN activity tracker may be more accurate. It should, however, be kept in mind that accelerometers have been shown to be less accurate in correctly classifying MVPA in children during free-living conditions when using a cut-point based on steps per minute [30].

Another limitation is that we did not distinguish between light physical activity and inactive time, which may have led to an overestimation of sedentary time. Furthermore, the time for automatic screen lock was included in the total screen time. Although we do not believe that this time is of significance for the study results, it is something that should be taken into consideration in future studies involving smartphone screen time. Moreover, we cannot rule out the possibility of a potential selection bias. It is possible that parents who had a specific interest in physical activity and screen time were more willing to participate in the study than parents with no interest in the subject were. Lastly, the relatively small sample size, when dividing the children and adolescents into groups according to gender and age, is another limitation.

Our society is becoming more and more digitized. The body of research using new technologies, such as smartphones, to measure health in adults is increasing. Nevertheless, few studies using smartphones have been conducted among children and adolescents. As unhealthy behaviours have a tendency to persist into adulthood, it is of great importance to study these behaviours in an early stage of life. Using smartphone applications as tools to identify and measure behaviours in children and adolescents is relevant in both research and clinical care. This study, measuring screen time objectively, is a first step in the direction of including new technologies in research conducted among children and adolescents. Objectively measured screen time may lead to better understanding of children and adolescents’ sedentary behaviour, which is needed to be able to promote and establish healthy activity patterns.

## Conclusion

This study is likely the first to objectively measure smartphone screen time in children and adolescents. The findings demonstrated no association between smartphone screen time and physical activity among children and adolescents between 10–15 years old. However, girls aged 14–15 years had both higher amount of screen time and were more physically active, compared to girls aged 10–12 years. Furthermore, boys reported significantly more time spent on computer and video games than girls did.

## Supporting information

S1 Table Demographic details of the municipalities for the schools included in this study. (DOCX)

S2 Table Parental questionnaire in English and Swedish. (DOCX)

S3 Table Child questionnaire in English and Swedish. (DOCX)

S1 File (DOCX)
