# Supplementary material for: Screen time and physical activity in children and adolescents aged 10–15 years
Source: PLoS One. 2021 Jul 9;16(7):e0254255. doi: 10.1371/journal.pone.0254255 (PMC8270173; doi:10.1371/journal.pone.0254255)
Supplement: S1 Table — (DOCX) [file pone.0254255.s001.docx]

# Supporting information

| **S1 Table.** Demographic details of the municipalities for the participating schools, according to Statistics Sweden and the Public Health Agency of Sweden, 2019. | | | |
| --- | --- | --- | --- |
|  |  | **Tyresö** ^a^ | **Botkyrka** ^b^ |
| **Number of residents** [1] | | 48 240 | 94 247 |
|  |  | **%** | **%** |
| **Foreign background** [1] | | 23 | 60 |
| **Obesity prevalence (BMI >30 m/kg^2^)** [2] | | 13 | 20 |
| **Daily smoking** [2] | | 6 | 10 |
| **Education level** [2] | |  |  |
|  | Elementary school | 11.2 | 19.8 |
|  | High school | 44.8 | 41.0 |
|  | University/college | 41.8 | 33.9 |
| BMI=Body Mass Index  ^a^ high socioeconomic status  ^b^ low socioeconomic status | |  |  |

**References**

1. Statistics Sweden [In Swedish: Statistiska centralbyrån]. Statistics database [In Swedish: Statistikdatabasen] [Internet]. Stockholm. Statistiska centralbyrån; 2019. [cited 2021 Jan 19]. Available [In Swedish] from: http://www.statistikdatabasen.scb.se/pxweb/sv/ssd/START__BE__BE0101__BE0101A/
2. Public Health Agency of Sweden [In Swedish: Folkhälsomyndigheten]. Municipality datasheet [In Swedish: Kommunfaktablad] [Internet]. Stockholm: Folkhälsomyndigheten. 2019. [cited 2021 Jan 19]. Available [In Swedish] from: https://www.folkhalsomyndigheten.se/kommunfakta/
