# Supplementary material for: Screen time and physical activity in children and adolescents aged 10–15 years
Source: PLoS One. 2021 Jul 9;16(7):e0254255. doi: 10.1371/journal.pone.0254255 (PMC8270173; doi:10.1371/journal.pone.0254255)
Supplement: S2 Table — (DOCX) [file pone.0254255.s002.docx]

| 1. **Background questions children** | |
| --- | --- |
| 1.1 | What year were you born?  ________ |
| 1.2 | Are you a girl or a boy:   - Girl - Boy |
| 1.3 | Enter your height in cm:  (roll-list with height in cm/do not know/do not want to answer) |
| 1.4 | Enter your weight in kg:  (roll-list with weight in kg /do not know/do not want to answer) |
| 1. **Questions about physical activity** | |
| 2.1 | How do you usually go to and from school?   - I walk all the way to school - I walk at least 5 minutes to the bus, metro or train - I bicycle all the way to school - I bicycle at least 5 minutes to the bus, metro or train - I go by car, bus, metro or train all the way |
| 2.2 | How long does it take? (fill in minutes e.g. 15 minutes/day)  _____ minutes/day on the way to school (open number)  _____ minutes/day on the way home back from school (open number) |
| 2.3 | How many times do you attend to physical education (PE) at school each week?   - Once - 2 times/week - 3 times/week - None - I do not have PE |
| 2.4 | Are you going to any organized sports or activity outside school?   - No - Yes, once a week - Yes, 1-2 times/week - Yes, 2-3 times/week - Yes, 3-4 times/week - Yes, 4-5 times/week - Yes, 5-6 times/week - Yes, every day |
| 2.5 | How physically active are you after school or in the evening a **regular school day** (walk, dance, gym, sports, other similar activity)   - Not active at all, most sedentary - Less than 30 minutes - 30-60 minutes - 1-2 hours - 2-3 hours - More than 3 hours - Do not know/do not want to answer |
| 2.6 | How physically active are you usually during a **regular weekend day** (Saturday & Sunday)? (e.g. walking, dancing, gymnastics, sports, other similar activity)   - Not active at all, most sedentary - Less than 30 minutes - 30-60 minutes - 1-2 hours - 2-3 hours - More than 3 hours - Do not know/do not want to answer |
| 2.7 | Would you like to be more physically active on the days than you are right now?   - Yes, I would like to be more physically active - No, I'm satisfied with my physical activity level - Do not know/do not want to answer |

| 1. **Questions about sleep** | |
| --- | --- |
| 3.1 | When do you usually get up from bed on a **normal school day**?   - Earlier than 06:00 - 06:00-06:30 - 06:30-07:00 - 07:00-07:30 - 07:30-08:00 - 08:00-08:30 - 08:30-09:00 - 09:00-09:30 - Later than at 09:30   When do you usually go to sleep a **normal school day**?   - Earlier than at 21:00 - 21:00-21:30 - 21:30-22:00 - 22:00-22:30 - 22:30-23:00 - 23:00-23:30 - Later than at 23:30 |
| 3.2 | When do you usually get up from bed on the **weekend** (Saturday and Sunday)?   - Earlier than at 07:00 - 07:00-07:30 - 07:30-08:00 - 08:00-08:30 - 08:30-09:00 - 09:00-09:30 - 09:30-10:00 - 10:00-10:30 - Later than at 10:30   When do you usually go to sleep on **the weekend** (Saturday and Sunday)?   - Earlier than at 21:00 - 21:00-21:30 - 21:30-22:00 - 22:00-22:30 - 22:30-23:00 - 23:00-23:30 - Later than at 23:30 |
| 3.3 | Would you like to change your daily screen time (i.e. smartphone, computer, TV or other) or are you satisfied with the way it is now?   - I would like to reduce my screen time - I’m satisfied - I want more screen time - Do not know/do not want to answer |

| **4. Questions about screen time and sedentary time** | | | | | | | | | |
| --- | --- | --- | --- | --- | --- | --- | --- | --- | --- |
| How much time do you spend on the following during **a normal weekday**, from the time you wake up until you go to bed? | | | | | | | | | |
|  | None | 15 min. or more | 30 min. | 1  hr | 2  hrs | 3  hrs | 4  hrs | 5  hrs | 6 hrs or more |
| 1. On the smartphone and/or tablet | □ | □ | □ | □ | □ | □ | □ | □ | □ |
| 1. Watching TV (including Netflix and YouTube) | □ | □ | □ | □ | □ | □ | □ | □ | □ |
| 1. Playing computer or video games | □ | □ | □ | □ | □ | □ | □ | □ | □ |
| 1. Sitting listening to music (e.g. Spotify/CD) | □ | □ | □ | □ | □ | □ | □ | □ | □ |
| 1. Sitting talking on the phone | □ | □ | □ | □ | □ | □ | □ | □ | □ |
| 1. Doing paper work or computer work (office work, email, paying bills etc.) | □ | □ | □ | □ | □ | □ | □ | □ | □ |
| 1. Sitting reading a book or magazine | □ | □ | □ | □ | □ | □ | □ | □ | □ |
| 1. Playing a musical instrument | □ | □ | □ | □ | □ | □ | □ | □ | □ |
| 1. Doing artwork or crafts (e.g. sewing, knitting, painting) | □ | □ | □ | □ | □ | □ | □ | □ | □ |
| 1. Going by car, bus, train or other motorized vehicle | □ | □ | □ | □ | □ | □ | □ | □ | □ |

| How much time do you spend on the following during **a normal weekend**, from the time you wake up to the time you go to bed? | | | | | | | | | |
| --- | --- | --- | --- | --- | --- | --- | --- | --- | --- |
|  | None | 15 min. or more | 30 min. | 1  hrs | 2  hrs | 3  hrs | 4  hrs | 5  hrs | 6 hrs or more |
| 1. On the smartphone and/or tablet | □ | □ | □ | □ | □ | □ | □ | □ | □ |
| 1. Watching TV (including Netflix and YouTube) | □ | □ | □ | □ | □ | □ | □ | □ | □ |
| 1. Playing computer or video games | □ | □ | □ | □ | □ | □ | □ | □ | □ |
| 1. Sitting listening to music (e.g. Spotify/CD) | □ | □ | □ | □ | □ | □ | □ | □ | □ |
| 1. Sitting talking on the phone | □ | □ | □ | □ | □ | □ | □ | □ | □ |
| 1. Doing paper work or computer work (office work, email, paying bills etc.) | □ | □ | □ | □ | □ | □ | □ | □ | □ |
| 1. Sitting reading a book or magazine | □ | □ | □ | □ | □ | □ | □ | □ | □ |
| 1. Playing a musical instrument | □ | □ | □ | □ | □ | □ | □ | □ | □ |
| 1. Doing artwork or crafts (e.g. sewing, knitting, painting) | □ | □ | □ | □ | □ | □ | □ | □ | □ |
| 1. Going by car, bus, train or other motorized vehicle | □ | □ | □ | □ | □ | □ | □ | □ | □ |

| **Children’s Questionnaire – Quality of life**  (Kid- and Kiddo-KINDL^R^, Questions for children) | | | | | | |
| --- | --- | --- | --- | --- | --- | --- |
| 1. **First of all, we would like know something about your physical health...** | | | | | | |
| During the past week… | | never | seldom | some-times | often | all the time |
| 1. … I felt ill | |  |  |  |  |  |
| 1. … I had a headache or tummy-ache | |  |  |  |  |  |
| 1. … I was tired and worn-out | |  |  |  |  |  |
| 1. … I felt strong and full of energy | |  |  |  |  |  |
|  |  | | | | | |
| 1. **…then something about how you’ve been feeling in general...** | | | | | | |
| During the past week… | | never | seldom | some-times | often | all the time |
| 1. … I had fun and laughed a lot | |  |  |  |  |  |
| 1. … I was bored | |  |  |  |  |  |
| 1. … I felt alone | |  |  |  |  |  |
| 1. … I was scared | |  |  |  |  |  |
|  |  | | | | | |
| 1. **… and how you have been feeling about yourself.** | | | | | | |
| During the past week… | | never | seldom | some-times | often | all the time |
| 1. … I was proud of myself | |  |  |  |  |  |
| 1. … I felt on the top of the world | |  |  |  |  |  |
| 1. … I felt pleased with myself | |  |  |  |  |  |
| 1. … I had lots of good ideas | |  |  |  |  |  |
|  | |  |  |  |  |  |
| **4. The next questions are about your family ...** | | | | | | |
| During the past week… | | never | seldom | some-times | often | all the time |
| 1. … I got on well with my parents | |  |  |  |  |  |
| 1. … I felt fine at home | |  |  |  |  |  |
| 1. … we quarrelled at home | |  |  |  |  |  |
| 1. … my parents stopped me from doing certain things | |  |  |  |  |  |
| **5. ... and then about friends.** | | | | | | |
| During the past week… | | never | seldom | some-times | often | all the time |
| 1. … I played with friends | |  |  |  |  |  |
| 1. … other kids liked me | |  |  |  |  |  |
| 1. … I got along well with my friends | |  |  |  |  |  |
| 1. … I felt different from other children | |  |  |  |  |  |
| **6. Last of all, we would like to know something about school.** | |  |  |  |  |  |
| During the last week in which I was at school... | | never | seldom | some-times | often | all the time |
| 1. … doing my schoolwork was easy | |  |  |  |  |  |
| 1. … I enjoyed my lessons | |  |  |  |  |  |
| 1. … I worried about my future | |  |  |  |  |  |
| 1. …I worried about bad marks or grades | |  |  |  |  |  |
|  | |  |  |  |  |  |
| © Kid- & Kiddo-KINDLR / parents / Swedish / Ravens-Sieberer & Bullinger / 2000 / page 3 | | | | | | |

| **1. Bakgrundsfrågor barn** | |
| --- | --- |
| 1.1 | Vilket år är du född?  ________ |
| 1.2 | Är du flicka eller pojke?   - Flicka - Pojke |
| 1.3 | Hur lång är du?  (vet ej/vill inte svara samt rullista med längd i cm) |
| 1.4 | Hur mycket väger du?  (vet ej/vill inte svara samt rullista med vikt i kg) |
| **2. Frågor om fysisk aktivitet** | |
| 2.1 | Hur tar du dig vanligtvis till och från skolan?   - Jag promenerar till skolan - Jag promenerar till buss, tunnelbana eller tåg som går till skolan (minst 5 minuter) - Jag cyklar till skolan - Jag cyklar till buss, tunnelbana eller tåg som går till skolan (minst 5 minuter) - Jag åker bil eller buss, tunnelbana, tåg hela vägen till skolan |
| 2.2 | Hur lång tid tar det? (fyll i antal minuter, exempelvis 15 minuter/dag)  _____ minuter/dag på väg till skolan (fri text)  _____ minuter/dag på väg hem (fri text) |
| 2.3 | Hur många idrottslektioner är du med på i skolan per vecka?   - 1 gång/vecka - 2 ggr/vecka - 3 ggr/vecka - ingen - har ej skolidrott |
| 2.4 | Går du på någon organiserad idrotts- eller sportaktivitet utanför skolan?   - Nej - Ja, 1 gång/vecka - Ja, 1-2 ggr/vecka - Ja, 2-3 ggr/vecka - Ja, 3-4 ggr/vecka - Ja, 4-5 ggr/vecka - Ja, 5-6 ggr/vecka - Ja, alla dagar |
| 2.5 | Hur fysiskt aktiv är du på kvällarna efter en **vanlig skoldag** (promenad, dans, gympa, idrott, annan motsvarande aktivitet)?   - ej aktiv alls, mest stillasittande - mindre än 30 minuter - 30-60 minuter - 1-2 timmar - 2-3 timmar - mer än 3 timmar per kväll - vet ej/vill inte svara |
| 2.6 | Hur fysisk aktiv är du i genomsnitt en **vanlig helgdag** (lördag & söndag) (t ex promenad, dans, gympa, idrott, annan motsvarande aktivitet)   - ej aktiv alls, mest stillasittande - mindre än 30 minuter - 30-60 minuter - 1-2 timmar - 2-3 timmar - mer än 3 timmar per dag - Vet ej/vill inte svara |
| 2.7 | Skulle du vilja vara mer fysiskt aktiv på dagarna än vad du är just nu?   - Jag skulle vilja vara mer fysiskt aktiv - Jag är nöjd med det jag gör just nu - Vet ej/vill inte svara |

| **3. Sömn** | |
| --- | --- |
| 3.1 | När går du upp i genomsnitt en **vanlig skoldag**?   - tidigare än 06:00 - 06:00-06:30 - 06:30-07:00 - 07:00-07:30 - 07:30-08:00 - 08:00-08:30 - 08:30-09:00 - 09:00-09:30 - Senare än 09:30   När går du och lägger dig i genomsnitt en **vanlig skoldag**?   - tidigare än 21:00 - 21:00-21:30 - 21:30-22:00 - 22:00-22:30 - 22:30-23:00 - 23:00-23:30 - Senare än 23:00 |
| 3.2 | När går du upp i genomsnitt en **vanlig helgdag** (lördag & söndag)?   - tidigare än 07:00 - 07:00-07:30 - 07:30-08:00 - 08:00-08:30 - 08:30-09:00 - 09:00-09:30 - 09:30-10:00 - 10:00-10:30 - Senare än 10:30   När går du och lägger dig i genomsnitt en **vanlig helgdag** (lördag & söndag)?   - tidigare än 21:00 - 21:00-21:30 - 21:30-22:00 - 22:00-22:30 - 22:30-23:00 - 23:00-23:30 - senare än 23:30 |

| 3.3 | Skulle du vilja minska din dagliga skärmtid (dvs surf och spel på mobil, dator, tv eller annat) eller känner du dig nöjd som det är?   - Jag skulle vilja minska min skärmtid - Jag är nöjd - Jag vill ha mer skärmtid - Vet ej/vill inte svara |
| --- | --- |

| **4. Frågor om skärmtid och stillasittande** | | | | | | | | | |
| --- | --- | --- | --- | --- | --- | --- | --- | --- | --- |
| Hur mycket tid spenderar du på följande under **en vanlig vardag**, från det att du vaknar till dess att du går och lägger dig? | | | | | | | | | |
|  | Inget | 15 min eller mer | 30 min | 1  tim | 2  tim | 3  tim | 4  tim | 5  tim | 6 tim eller mer |
| 1. Surfa på mobil och/eller läsplatta | □ | □ | □ | □ | □ | □ | □ | □ | □ |
| 1. Titta på TV (inklusive tjänster som Netflix, Youtube mm) | □ | □ | □ | □ | □ | □ | □ | □ | □ |
| 1. Spela dator- eller TV-spel | □ | □ | □ | □ | □ | □ | □ | □ | □ |
| 1. Sitta ned och lyssna på musik på Spotify, cd eller annat | □ | □ | □ | □ | □ | □ | □ | □ | □ |
| 1. Sitta ned och prata i telefon | □ | □ | □ | □ | □ | □ | □ | □ | □ |
| 1. Pappersarbete eller motsvarande på datorn (kontorsarbete, svara på mail, betala räkningar) | □ | □ | □ | □ | □ | □ | □ | □ | □ |
| 1. Läsa en bok eller tidskrift | □ | □ | □ | □ | □ | □ | □ | □ | □ |
| 1. Spela ett musikinstrument | □ | □ | □ | □ | □ | □ | □ | □ | □ |
| 1. Konst och hantverk, t ex sy, sticka, måla, pyssla mm | □ | □ | □ | □ | □ | □ | □ | □ | □ |
| 1. Åka bil, buss, tåg eller annat motordrivet fordon | □ | □ | □ | □ | □ | □ | □ | □ | □ |

| Hur mycket tid spenderar du på följande under **en vanlig helgdag**, från det att du vaknar till dess att du går och lägger dig? | | | | | | | | | |
| --- | --- | --- | --- | --- | --- | --- | --- | --- | --- |
|  | Inget | 15 min eller mer | 30 min | 1  tim | 2  tim | 3  tim | 4  tim | 5  tim | 6 tim eller mer |
| 1. Surfa på mobil och/eller läsplatta | □ | □ | □ | □ | □ | □ | □ | □ | □ |
| 1. Titta på TV (inklusive tjänster som Netflix, Youtube mm) | □ | □ | □ | □ | □ | □ | □ | □ | □ |
| 1. Spela dator- eller TV-spel | □ | □ | □ | □ | □ | □ | □ | □ | □ |
| 1. Sitta ned och lyssna på musik på Spotify, cd eller annat | □ | □ | □ | □ | □ | □ | □ | □ | □ |
| 1. Sitta ned och prata i telefon | □ | □ | □ | □ | □ | □ | □ | □ | □ |
| 1. Pappersarbete eller motsvarande på datorn (kontorsarbete, svara på mail, betala räkningar) | □ | □ | □ | □ | □ | □ | □ | □ | □ |
| 1. Läsa en bok eller tidskrift | □ | □ | □ | □ | □ | □ | □ | □ | □ |
| 1. Spela ett musikinstrument | □ | □ | □ | □ | □ | □ | □ | □ | □ |
| 1. Konst och hantverk, t ex sy, sticka, måla, pyssla mm | □ | □ | □ | □ | □ | □ | □ | □ | □ |
| 1. Åka bil, buss, tåg eller annat motordrivet fordon | □ | □ | □ | □ | □ | □ | □ | □ | □ |

| **Frågeformulär - barns livskvalitet**  (Kid- och Kiddo-KINDL^R^, frågor för barn) | | | | | | |
| --- | --- | --- | --- | --- | --- | --- |
| 1. **Först skulle vi vilja veta lite om din kropp…** | | | | | | |
| Förra veckan… | | aldrig | sällan | ibland | ofta | alltid |
| 1. … kände jag mig sjuk | |  |  |  |  |  |
| 1. … hade jag ont i huvudet eller ont i magen | |  |  |  |  |  |
| 1. … kände jag mig trött eller hängig | |  |  |  |  |  |
| 1. … var jag full av energi och uthållighet | |  |  |  |  |  |
|  |  | | | | | |
| 1. **sedan lite om hur du känner dig …** | | | | | | |
| Förra veckan… | | aldrig | sällan | ibland | ofta | alltid |
| 1. … skrattade jag och hade roligt | |  |  |  |  |  |
| 1. … hade jag långtråkigt | |  |  |  |  |  |
| 1. … kände jag mig ensam | |  |  |  |  |  |
| 1. … var jag rädd | |  |  |  |  |  |
| 1. **… och vad du tycker om dig själv.** | | | | | | |
| Förra veckan… | | aldrig | sällan | ibland | ofta | alltid |
| 1. … var jag stolt över mig själv | |  |  |  |  |  |
| 1. … tyckte jag bra om mig själv | |  |  |  |  |  |
| 1. … gillade jag mig själv | |  |  |  |  |  |
| 1. … hade jag massor av roliga idéer | |  |  |  |  |  |
|  | |  |  |  |  |  |
| **4. I frågorna som kommer sedan handlar det om din familj …** | | | | | | |
| Förra veckan… | | aldrig | sällan | ibland | ofta | alltid |
| 1. … kom jag bra överens med mina föräldrar | |  |  |  |  |  |
| 1. … tyckte jag att det var roligt hemma | |  |  |  |  |  |
| 1. … bråkade vi mycket hemma | |  |  |  |  |  |
| 1. … förbjöd mig mina föräldrar att göra olika saker | |  |  |  |  |  |

| **5. …och sedan om dina kompisar.** | | | | | |
| --- | --- | --- | --- | --- | --- |
| Förra veckan… | aldrig | sällan | ibland | ofta | alltid |
| 1. … gjorde jag saker med mina kompisar |  |  |  |  |  |
| 1. … tyckte de andra kompisarna om mig |  |  |  |  |  |
| 1. … kom jag bra överens med mina kompisar |  |  |  |  |  |
| 1. … hade jag känslan att jag var annorlunda än de andra |  |  |  |  |  |
| **6. Nu skulle vi vilja veta lite om skolan.** |  |  |  |  |  |
| När jag var i skolan förra veckan… | aldrig | sällan | ibland | ofta | alltid |
| 1. … klarade jag av skoluppgifterna bra |  |  |  |  |  |
| 1. … tyckte jag att det var roligt att lära mig saker |  |  |  |  |  |
| 1. … var jag orolig för framtiden |  |  |  |  |  |
| 1. … var jag rädd för att få dåliga betyg |  |  |  |  |  |
|  |  |  |  |  |  |
|  |  |  |  |  |  |
| © Kid- & Kiddo-KINDLR / parents / Swedish / Ravens-Sieberer & Bullinger / 2000 / page 3 | | | | | |
