# Supplementary material for: Screen time and physical activity in children and adolescents aged 10–15 years
Source: PLoS One. 2021 Jul 9;16(7):e0254255. doi: 10.1371/journal.pone.0254255 (PMC8270173; doi:10.1371/journal.pone.0254255)
Supplement: S3 Table — (DOCX) [file pone.0254255.s003.docx]

| **1.Background questions parents** | |
| --- | --- |
| 1.1 | What year were you born?  ________  What year is your child participating in this research study born?  ________ |
| 1.2 | What is your sex?   - Woman - Man |
| 1.3 | In which country were you born?   - Sweden or another Nordic country - Europe - Another country outside Europe |
| 1.4 | Enter your height in cm:  (roll-list with height in cm/Do not know/Do not want to answer) |
| 1.5 | Enter your weight in kg:  (roll-list with weight in kg /Do not know/Do not want to answer) |
| 1.6 | What is your highest education?   - Elementary school - 2-year high school/vocational school - 3-year high school - University - Do not know/Do not want to answer |

| **2. Questions about your physical activity level** | | | | | | | | | | | | |
| --- | --- | --- | --- | --- | --- | --- | --- | --- | --- | --- | --- | --- |
|  |  | | | | | | | | | | | |
| 2.1 | What is your daily activity level (work, studies or similar)? | | | | | | | | | | | |
|  | Sitting most of the time | |  | | Standing or walking most of the time | |  | | Heavy labour |  | Do not know/ Do not want to answer | |
|  | 1 | | 2 | | 3 | | 4 | | 5 | 6 | 7 | |
| 2.2 | What is your usual physical activity level during leisure time? | | | | | | | | | | | |
|  | Sitting most of the time |  | | Walking 30 minutes per day | |  | | Vigorous activity 60 minutes per day | |  | | Do not know/ Do not want to answer |
|  | 1 | 2 | | 3 | | 4 | | 5 | | 6 | | 7 |
| 2.3 | How many hours do you usually sit during a normal day? (do not count sleep)   - Never - 1-3 hours - 4-6 hours - 7-9 hours - 10-12 hours - 13-15 hours - Almost the entire day - Do not know/Do not want to answer | | | | | | | | | | | |
| 2.4 | Do you exercise or do sports regularly?   - Yes - No - Do not know/Do not want to answer | | | | | | | | | | | |
|  | | | | | | | | | | | | |

| **4. Questions about screen time and sedentary time** | | | | | | | | | |
| --- | --- | --- | --- | --- | --- | --- | --- | --- | --- |
| How much time do you spend on the following during **a normal weekday**, from the time you wake up until you go to bed? | | | | | | | | | |
|  | None | 15 min. or more | 30 min. | 1  hr | 2  hrs | 3  hrs | 4  hrs | 5  hrs | 6 hrs or more |
| 1. On the smartphone and/or tablet | □ | □ | □ | □ | □ | □ | □ | □ | □ |
| 1. Watching TV (including Netflix and YouTube) | □ | □ | □ | □ | □ | □ | □ | □ | □ |
| 1. Playing computer or video games | □ | □ | □ | □ | □ | □ | □ | □ | □ |
| 1. Sitting listening to music (e.g. Spotify/CD) | □ | □ | □ | □ | □ | □ | □ | □ | □ |
| 1. Sitting talking on the phone | □ | □ | □ | □ | □ | □ | □ | □ | □ |
| 1. Doing paper work or computer work (office work, email, paying bills etc.) | □ | □ | □ | □ | □ | □ | □ | □ | □ |
| 1. Sitting reading a book or magazine | □ | □ | □ | □ | □ | □ | □ | □ | □ |
| 1. Playing a musical instrument | □ | □ | □ | □ | □ | □ | □ | □ | □ |
| 1. Doing artwork or crafts (e.g. sewing, knitting, painting) | □ | □ | □ | □ | □ | □ | □ | □ | □ |
| 1. Driving a car, bus, train or other motorized vehicle | □ | □ | □ | □ | □ | □ | □ | □ | □ |

| How much time do you spend on the following during **a normal weekend**, from the time you wake up to the time you go to bed? | | | | | | | | | |
| --- | --- | --- | --- | --- | --- | --- | --- | --- | --- |
|  | None | 15 min. or more | 30 min. | 1  hrs | 2  hrs | 3  hrs | 4  hrs | 5  hrs | 6 hrs or more |
| 1. On the smartphone and/or tablet | □ | □ | □ | □ | □ | □ | □ | □ | □ |
| 1. Watching TV (including Netflix and YouTube) | □ | □ | □ | □ | □ | □ | □ | □ | □ |
| 1. Playing computer or video games | □ | □ | □ | □ | □ | □ | □ | □ | □ |
| 1. Sitting listening to music (e.g. Spotify/CD) | □ | □ | □ | □ | □ | □ | □ | □ | □ |
| 1. Sitting talking on the phone | □ | □ | □ | □ | □ | □ | □ | □ | □ |
| 1. Doing paper work or computer work (office work, email, paying bills etc.) | □ | □ | □ | □ | □ | □ | □ | □ | □ |
| 1. Sitting reading a book or magazine | □ | □ | □ | □ | □ | □ | □ | □ | □ |
| 1. Playing a musical instrument | □ | □ | □ | □ | □ | □ | □ | □ | □ |
| 1. Doing artwork or crafts (e.g. sewing, knitting, painting) | □ | □ | □ | □ | □ | □ | □ | □ | □ |
| 1. Driving a car, bus, train or other motorized vehicle | □ | □ | □ | □ | □ | □ | □ | □ | □ |

| **1.Bakgrundsfrågor förälder** | |
| --- | --- |
| 1.1 | Vilket år är du född?  ________  Vilket år är ditt barn som ska vara med i forskningsstudien född?  ________ |
| 1.2 | Är du man eller kvinna?   - Kvinna - Man |
| 1.3 | I vilket land är du född?   - Sverige eller annat land i Norden - Europa - Annat land utanför Europa |
| 1.4 | Hur lång är du?  (rullistan med vet ej/vill inte svara samt längd i cm) |
| 1.5 | Hur mycket väger du?  (rullista med vet ej/vill inte svara samt vikt i kg) |
| 1.6 | Hur många års utbildning har du?   - Grundskola/realskola - 2-årig gymnasieskola/fackskola - 3-årig gymnasieskola - Universitet/Högskola - Vet ej/vill inte svara |

| **2. Frågor om din fysiska aktivitet** | | | | | | | | | | | | |
| --- | --- | --- | --- | --- | --- | --- | --- | --- | --- | --- | --- | --- |
|  |  | | | | | | | | | | | |
| 2.1 | Vilken aktivitetsnivå har du vanligtvis i din dagliga sysselsättning (jobb, studier eller motsvarande)? | | | | | | | | | | | |
|  | Sitter mest | |  | | Står och går mest | |  | | Tungt kroppsarbete |  | Vet ej/Vill inte svara | |
|  | 1 | | 2 | | 3 | | 4 | | 5 | 6 | 7 | |
| 2.2 | Vilken aktivitetsnivå har du vanligtvis på fritiden? | | | | | | | | | | | |
|  | Sitter mest |  | | Promenerar 30 minuter per dag | |  | | Ansträngande aktivitet 60 minuter/dag | |  | | Vet ej/vill inte svara |
|  | 1 | 2 | | 3 | | 4 | | 5 | | 6 | | 7 |
| 2.3 | Hur mycket sitter du under ett normalt dygn om man räknar bort sömn?   - aldrig - 1-3 timmar - 4-6 timmar - 7-9 timmar - 10-12 timmar - 13-15 timmar - så gott som hela dagen - Vet ej/vill inte svara | | | | | | | | | | | |
| 2.4 | Tränar eller sportar du regelbundet?   - Ja - Nej - Vet ej/ vill inte svara | | | | | | | | | | | |

| **3. Frågor om din skärmtid och stillasittande** |
| --- |
|  |

| Hur mycket tid spenderar du på följande under **en vanlig vardag**, från det att du vaknar till dess att du går och lägger dig? | | | | | | | | | |
| --- | --- | --- | --- | --- | --- | --- | --- | --- | --- |
|  | Inget | 15 min eller mer | 30 min | 1  tim | 2  tim | 3  tim | 4  tim | 5  tim | 6 tim eller mer |
| 1. Surfa på mobil och/eller läsplatta | □ | □ | □ | □ | □ | □ | □ | □ | □ |
| 1. Titta på TV (inklusive tjänster som Netflix, Youtube mm) | □ | □ | □ | □ | □ | □ | □ | □ | □ |
| 1. Spela dator- eller TV-spel | □ | □ | □ | □ | □ | □ | □ | □ | □ |
| 1. Sitta ned och lyssna på musik på Spotify, cd eller annat | □ | □ | □ | □ | □ | □ | □ | □ | □ |
| 1. Sitta ned och prata i telefon | □ | □ | □ | □ | □ | □ | □ | □ | □ |
| 1. Pappersarbete eller motsvarande på datorn (kontorsarbete, svara på mail, betala räkningar) | □ | □ | □ | □ | □ | □ | □ | □ | □ |
| 1. Läsa en bok eller tidskrift | □ | □ | □ | □ | □ | □ | □ | □ | □ |
| 1. Spela ett musikinstrument | □ | □ | □ | □ | □ | □ | □ | □ | □ |
| 1. Konst och hantverk, t ex sy, sticka, måla, pyssla mm | □ | □ | □ | □ | □ | □ | □ | □ | □ |
| 1. Kör bil, buss, tåg eller annat motordrivet fordon | □ | □ | □ | □ | □ | □ | □ | □ | □ |

| Hur mycket tid spenderar du på följande under **en vanlig helgdag**, från det att du vaknar till dess att du går och lägger dig? | | | | | | | | | |
| --- | --- | --- | --- | --- | --- | --- | --- | --- | --- |
|  | Inget | 15 min eller mer | 30 min | 1  tim | 2  tim | 3  tim | 4  tim | 5  tim | 6 tim eller mer |
| 1. Surfa på mobil och/eller läsplatta | □ | □ | □ | □ | □ | □ | □ | □ | □ |
| 1. Titta på TV (inklusive tjänster som Netflix, Youtube mm) | □ | □ | □ | □ | □ | □ | □ | □ | □ |
| 1. Spela dator- eller TV-spel | □ | □ | □ | □ | □ | □ | □ | □ | □ |
| 1. Sitta ned och lyssna på musik på Spotify, cd eller annat | □ | □ | □ | □ | □ | □ | □ | □ | □ |
| 1. Sitta ned och prata i telefon | □ | □ | □ | □ | □ | □ | □ | □ | □ |
| 1. Pappersarbete eller motsvarande på datorn (kontorsarbete, svara på mail, betala räkningar) | □ | □ | □ | □ | □ | □ | □ | □ | □ |
| 1. Läsa en bok eller tidskrift | □ | □ | □ | □ | □ | □ | □ | □ | □ |
| 1. Spela ett musikinstrument | □ | □ | □ | □ | □ | □ | □ | □ | □ |
| 1. Konst och hantverk, t ex sy, sticka, måla, pyssla mm | □ | □ | □ | □ | □ | □ | □ | □ | □ |
| 1. Kör bil, buss, tåg eller annat motordrivet fordon | □ | □ | □ | □ | □ | □ | □ | □ | □ |
