# Supplementary material for: Screen time and physical activity in children and adolescents aged 10–15 years
Source: PLoS One. 2021 Jul 9;16(7):e0254255. doi: 10.1371/journal.pone.0254255 (PMC8270173; doi:10.1371/journal.pone.0254255)
Supplement: S1 File — (DOCX) [file pone.0254255.s004.docx]

# CODEBOOK for the SCRIIN study, Karolinska Institutet, Sweden

| **Child/adolescent questionnaire** | | |
| --- | --- | --- |
| **Variable name** | **Description/Question** | **Value description** |
| consent | Web-based consent to participation | 1 = yes  2 = no |
|  | What you answer here will not your parents or school staff know. |  |
| date_of_birth | What year were you born? | open numbers |
| sex | Are you a girl or a boy? | 1 = girl  2 = boy |
| heigh_cm | Enter your height in cm | 1 = <130 cm  2 = 135  3 = 136 cm  and so on….  66 = 194  67 = 195  68 = >195 cm |
| weight_kg | Enter your weight in kg | 2 = don’t know/don’t want to answer  1 = > 30 kg  3 = 31  4 = 32  5 =33  6 = 34  And so on….  62 = 90 kg  63 = > 90 kg |
| trans_school | How do you usually go to and from school? | 1 = I walk all the way to school  2 = I walk at least 5 minutes to bus, metro or train  3 = I bicycle all the way to school  4 = I bicycle at least 5 minutes to bus, metro or train  5 = I go by car, bus, metro or train all the way |
| trans_time_school | How long does it take? (fill in minutes, e.g. 15 minutes/day)  minutes/day on the way to school | open number |
| trans_time_home | How long does it take? (fill in minutes, e.g. 15 minutes/day)  minutes/day on the way home | open number |
| sportlesson | How many times do you attend to physical education (PE) at school each week? | 1 = once a week  2 = 2 times/week  3 = 3 times/week  4 = none  5 = I do not have PE |
| act_org | Are you going to any organized sports or activity outside school? | 1 = no  2 = yes, once a week  3 = yes, 1-2 times/week  4 = yes, 2-3 times/week  5= yes, 3-4 times/week  6= yes, 4-5 times/week  7= yes, 5-6 times/week  8= yes, every day |
| pa_evening | How physically active are you after school or in the evening a regular school day (walk, dance, gym, sports, other similar activity)? | 1 = not active at all, most sedentary  2 = less than 30 minutes  3 = 30-60 minutes  4 = 1-2 hours  5 = 2-3 hours  6 = more than 3 hours  7= do not know/do not want to answer |
| pa_we | How physically active are you usually during a regular weekend day (Saturday & Sunday)? (e.g. walking, dancing, gymnastics, sports, other similar activity) | 1 = not active at all, most sedentary  2 = less than 30 minutes  3 = 30-60 minutes  4 = 1-2 hours  5 = 2-3 hours  6 = more than 3 hours  7= do not know/do not want to answer |
| pa_more | Would you like to be more physically active on the days than you are right now? | 1 = yes, I would like to be more physically active  2 = no, I'm satisfied with my physical activity level  3= do not know/do not want to answer |
| screen_fam | Do you and your family use screens at home during dinner (TV, tablet, computer, mobile, etc.)? | 1 = no, never  2 = yes, but only on weekends  3 = yes, 1-2 daily dinners/week  4 = yes, 3-4 daily dinners/week  5 = yes, 5-6 dinners/week  6 = yes, every dinner |
| screen_bed | Do you have access to screens (TV, tablet, mobile, computer, etc.) in your bedroom? | 1 = yes  2 = no  3 = do not know/do not want to answer |
| screen_sleep | Do you look at any type of screen before you go to sleep (mobile, TV, tablet, computer, etc.)? | 1 = no, never  2 = yes, sometimes  3 = yes, often/always |
| wakeup_wd | When do you usually get up from bed on a normal school day? | 1 = earlier than 06:00  2 = 06:00-06:30  3 =06:30-07:00  4 = 07:00-07:30  5 = 07:30-08:00  6 = 08:00-08:30  7 = 08:30-09:00  8 = 09:00-09:30  9 = later than 09:30 |
| bedtime_wd | When do you usually go to sleep a normal school day? | 1 = earlier than 21:00  2 = 21:00-21:30  3 = 21:20-22:00  4 = 22:00-22:30  5 = 22:30-23:00  6 = 23:00-23:30  7 = later than 23:30 |
| wakeup_we | When do you usually get upfrom bed on the weekend (Saturday & Sunday)? | 1 = earlier than 07:00  2 = 07:00-07:30  3 = 07:30-08:00  4 = 08:00-08:30  5 = 08:30-09:00  6 = 09:00-09:30  7 = 09:30-10:00  8 = 10:00-10:30  9 = later than 10:30 |
| bedtime_we | When do you usually go to sleep on the weekend (Saturday & Sunday)? | 1 = earlier than 21:00  2 = 21:00-21:30  3 = 21:20-22:00  4 = 22:00-22:30  5 = 22:30-23:00  6 = 23:00-23:30  7 = later than 23:30 |
| screen_change | Would you like to change your daily screen time (i.e. surf and games on mobile, computer, TV or other) or are you satisfied with the way it is now? | 1 = I would like to reduce my screen time  2 = I'm satisfied  3 = I want more screen time  4 = do not know/do not want to answer |
| mobile_wd | How much time do you spend on the following during a normal weekday, from the time you wake up until you go to bed?  On the smartphone and/or tablet | 1 = none  2 = 15 min or more  3 = 30 min  4 = 1 hour  5 = 2 hours  6 = 3 hours  7 = 4 hours  8 = 5 hours  9 = 6 hours or more |
| tv_wd | How much time do you spend on the following during a normal weekday, from the time you wake up until you go to bed?  Watch TV (including Netflix and Youtube) | 1 = none  2 = 15 min or more  3 = 30 min  4 = 1 hour  5 = 2 hours  6 = 3 hours  7 = 4 hours  8 = 5 hours  9 = 6 hours or more |
| game_wd | How much time do you spend on the following during a normal weekday, from the time you wake up until you go to bed?  Playing computer or video games | 1 = none  2 = 15 min or more  3 = 30 min  4 = 1 hour  5 = 2 hours  6 = 3 hours  7 = 4 hours  8 = 5 hours  9 = 6 hours or more |
| music_wd | How much time do you spend on the following during a normal weekday, from the time you wake up until you go to bed?  Sitting listening to music (e.g. Spotify/CD) | 1 = none  2 = 15 min or more  3 = 30 min  4 = 1 hour  5 = 2 hours  6 = 3 hours  7 = 4 hours  8 = 5 hours  9 = 6 hours or more |
| talk_wd | How much time do you spend on the following during a normal weekday, from the time you wake up until you go to bed?  Sitting and talking on the phone | 1 = none  2 = 15 min or more  3 = 30 min  4 = 1 hour  5 = 2 hours  6 = 3 hours  7 = 4 hours  8 = 5 hours  9 = 6 hours or more |
| paper_wd | How much time do you spend on the following during a normal weekday, from the time you wake up until you go to bed?  Doing paper work or computer work (office work, emails, paying bills etc.) | 1 = none  2 = 15 min or more  3 = 30 min  4 = 1 hour  5 = 2 hours  6 = 3 hours  7 = 4 hours  8 = 5 hours  9 = 6 hours or more |
| book_wd | How much time do you spend on the following during a normal weekday, from the time you wake up until you go to bed?  Sitting reading a book or magazine | 1 = none  2 = 15 min or more  3 = 30 min  4 = 1 hour  5 = 2 hours  6 = 3 hours  7 = 4 hours  8 = 5 hours  9 = 6 hours or more |
| instrument_wd | How much time do you spend on the following during a normal weekday, from the time you wake up until you go to bed?  Playing a musical instrument | 1 = none  2 = 15 min or more  3 = 30 min  4 = 1 hour  5 = 2 hours  6 = 3 hours  7 = 4 hours  8 = 5 hours  9 = 6 hours or more |
| art_wd | How much time do you spend on the following during a normal weekday, from the time you wake up until you go to bed?  Doing artwork or crafts (e.g. painting, knitting etc.) | 1 = none  2 = 15 min or more  3 = 30 min  4 = 1 hour  5 = 2 hours  6 = 3 hours  7 = 4 hours  8 = 5 hours  9 = 6 hours or more |
| car_wd | How much time do you spend on the following during a normal weekday, from the time you wake up until you go to bed?  Sitting and driving in a car, bus, train or other motorized vehicle | 1 = none  2 = 15 min or more  3 = 30 min  4 = 1 hour  5 = 2 hours  6 = 3 hours  7 = 4 hours  8 = 5 hours  9 = 6 hours or more |
| mobile_we | How much time do you spend on the following during a normal weekend, from the time you wake up until you go to bed?  On the smartphone and/or tablet | 1 = no time  2 = 15 min or more  3 = 30 min  4 = 1 hour  5 = 2 hours  6 = 3 hours  7 = 4 hours  8 = 5 hours  9 = 6 hours or more |
| tv_we | How much time do you spend on the following during a normal weekend, from the time you wake up until you go to bed?  Watch TV (including Netflix and Youtube) | 1 = none  2 = 15 min or more  3 = 30 min  4 = 1 hour  5 = 2 hours  6 = 3 hours  7 = 4 hours  8 = 5 hours  9 = 6 hours or more |
| game_we | How much time do you spend on the following during a normal weekend, from the time you wake up until you go to bed?  Playing computer or video games | 1 = none  2 = 15 min or more  3 = 30 min  4 = 1 hour  5 = 2 hours  6 = 3 hours  7 = 4 hours  8 = 5 hours  9 = 6 hours or more |
| music_we | How much time do you spend on the following during a normal weekend, from the time you wake up until you go to bed?  Sitting listening to music (e.g. Spotify/CD) | 1 = none  2 = 15 min or more  3 = 30 min  4 = 1 hour  5 = 2 hours  6 = 3 hours  7 = 4 hours  8 = 5 hours  9 = 6 hours or more |
| talk_we | How much time do you spend on the following during a normal weekend, from the time you wake up until you go to bed?  Sitting and talking on the phone | 1 = none  2 = 15 min or more  3 = 30 min  4 = 1 hour  5 = 2 hours  6 = 3 hours  7 = 4 hours  8 = 5 hours  9 = 6 hours or more |
| paper_we | How much time do you spend on the following during a normal weekend, from the time you wake up until you go to bed?  Doing paper work or computer work (office work, emails, paying bills etc.) | 1 = none  2 = 15 min or more  3 = 30 min  4 = 1 hour  5 = 2 hours  6 = 3 hours  7 = 4 hours  8 = 5 hours  9 = 6 hours or more |
| book_we | How much time do you spend on the following during a normal weekend, from the time you wake up until you go to bed?  Sitting reading a book or magazine | 1 = none  2 = 15 min or more  3 = 30 min  4 = 1 hour  5 = 2 hours  6 = 3 hours  7 = 4 hours  8 = 5 hours  9 = 6 hours or more |
| instrument_we | How much time do you spend on the following during a normal weekend, from the time you wake up until you go to bed?  Playing a musical instrument | 1 = none  2 = 15 min or more  3 = 30 min  4 = 1 hour  5 = 2 hours  6 = 3 hours  7 = 4 hours  8 = 5 hours  9 = 6 hours or more |
| art_we | How much time do you spend on the following during a normal weekend, from the time you wake up until you go to bed?  Doing artwork or crafts (e.g. painting, knitting etc.) | 1 = none  2 = 15 min or more  3 = 30 min  4 = 1 hour  5 = 2 hours  6 = 3 hours  7 = 4 hours  8 = 5 hours  9 = 6 hours or more |
| car_we | How much time do you spend on the following during a normal weekend, from the time you wake up until you go to bed?  Sitting and driving in a car, bus, train or other motorized vehicle | 1 = none  2 = 15 min or more  3 = 30 min  4 = 1 hour  5 = 2 hours  6 = 3 hours  7 = 4 hours  8 = 5 hours  9 = 6 hours or more |
| health_sick | First of all, we would like to know something about your physical health…  During the past week …  ... I felt ill | 1 = never  2 = seldom  3 = sometimes  4 = often  5 = all the time |
| health_pain | First of all, we would like to know something about your physical health…  During the past week …  ... I had a headache or tummy-ache | 1 = never  2 = seldom  3 = sometimes  4 = often  5 = all the time |
| health_tired | First of all, we would like to know something about your physical health…  During the past week …  ... I was tired and worn-out | 1 = never  2 = seldom  3 = sometimes  4 = often  5 = all the time |
| health_energy | First of all, we would like to know something about your physical health…  During the past week …  ...I felt strong and full of energy | 1 = never  2 = seldom  3 = sometimes  4 = often  5 = all the time |
| feel_fun | Then something about how you’ve been feeling in general…  During the past week …  ... I had fun and laughed a lot | 1 = never  2 = seldom  3 = sometimes  4 = often  5 = all the time |
| feel_bored | Then something about how you’ve been feeling in general…  During the past week …  ... I was bored | 1 = never  2 = seldom  3 = sometimes  4 = often  5 = all the time |
| feel_alone | Then something about how you’ve been feeling in general…  During the past week …  ... I felt alone | 1 = never  2 = seldom  3 = sometimes  4 = often  5 = all the time |
| feel_scared | Then something about how you’ve been feeling in general…  During the past week …  ... I was scared | 1 = never  2 = seldom  3 = sometimes  4 = often  5 = all the time |
| you_proud | ... and how you have been feeling about yourself  During the past week …  ... I was proud of myself | 1 = never  2 = seldom  3 = sometimes  4 = often  5 = all the time |
| you_good | ... and how you have been feeling about yourself  During the past week …  ... I felt on the top of the world | 1 = never  2 = seldom  3 = sometimes  4 = often  5 = all the time |
| you_like | ... and how you have been feeling about yourself  During the past week …  ... I felt pleased with myself | 1 = never  2 = seldom  3 = sometimes  4 = often  5 = all the time |
| you_fun | ... and how you have been feeling about yourself  During the past week …  ... I had lots of good ideas | 1 = never  2 = seldom  3 = sometimes  4 = often  5 = all the time |
| family_agree | The next questions are about your family ...  During the past week …  ... I got on well with my parents | 1 = never  2 = seldom  3 = sometimes  4 = often  5 = all the time |
| family_fun | The questions that come below are about your family ...  During the past week …  ... I felt fine at home | 1 = never  2 = seldom  3 = sometimes  4 = often  5 = all the time |
| family_angry | The questions that come below are about your family ...  During the past week …  ... We quarrelled at home | 1 = never  2 = seldom  3 = sometimes  4 = often  5 = all the time |
| family_forbid | The questions that come below are about your family ...  During the past week …  … My parents stopped me from doing certain things | 1 = never  2 = seldom  3 = sometimes  4 = often  5 = all the time |
| friend_social | ... and then about your friends  During the past week …  ... I played with friends | 1 = never  2 = seldom  3 = sometimes  4 = often  5 = all the time |
| friend_like | ... and then about your friends  During the past week …  ... Other kids liked me | 1 = never  2 = seldom  3 = sometimes  4 = often  5 = all the time |
| friend_agree | ... and then about your friends  During the past week …  ... I got along well with my friends | 1 = never  2 = seldom  3 = sometimes  4 = often  5 = all the time |
| friend_diff | ... and then about your friends  During the past week …  ... I felt different from other children | 1 = never  2 = seldom  3 = sometimes  4 = often  5 = all the time |
| school_task | Last of all, we would like to know something about school  During the last week in which I was in school…  ... doing my school work was easy | 1 = never  2 = seldom  3 = sometimes  4 = often  5 = all the time |
| school_learn | Now we want to know a little about the school  During the last week in which I was in school…  ... I enjoyed my lessons | 1 = never  2 = seldom  3 = sometimes  4 = often  5 = all the time |
| school_future | Now we want to know a little about the school  During the last week in which I was in school…  ... I worried about my future | 1 = never  2 = seldom  3 = sometimes  4 = often  5 = all the time |
| school_grades | Now we want to know a little about the school  During the last week in which I was in school…  ... I worried about bad marks or grades | 1 = never  2 = seldom  3 = sometimes  4 = often  5 = all the time |

| **Parental questionnaire** | | |
| --- | --- | --- |
| **Variable name** | **Description/Question** | **Value description** |
| consent_p | Web-based consent to participation | 1 = yes  2 = no |
| date_of_birth_p | What year were you born? | open numbers |
| date_of_birth_child_p | What year is your child (participating in this research study) born? | open numbers |
| sex_parent | What is your sex? | 1 = girl  2 = boy |
| country_p | In which country were you born? | 1 = Sweden or another Nordic country  2 = Europe  3 = Another country outside Europe |
| height_cm_p | Enter your height in cm | 1 = <145 cm  2 = 146  3 = 147 cm  and so on….  76 = 219  77 = 220  78 = >220 cm  79 = do not know/do not want to answer |
| weight_kg_p | Enter your weight in kg | 2 = > 40 kg  1 = 40 kg  3 = 41  4 = 42  5 =43  6 = 44  And so on….  198 = 235 kg  199 = > 235 kg  200 = do not know/do not want to answer |
| education_p | What is your highest education? | 1 = elementary school  2 = 2-year high school/vocational school  3 = 3-year high school  4 = university  5 = do not know/do not want to answer |
| pa_daily_p | What is your daily activity level (work, studies or similar)? | 1 = sitting most of the time  2  3 = standing and walking most of the time  4  5 = heavy labour  6  7 = do not know/do not want to answer |
| pa_leisure_p | What is your usual physical activity level during leisure time? | 1 = sitting most of the time  2  3 = walking 30 minutes per day  4  5 = vigorous activity 60 minutes per day  6  7 = do not know/do not want to answer |
| sitting_p | How many hours do you usually sit during a normal day? | 1 = never  2 = 1-3 hours  3 = 4-6 hours  4 = 7-9 hours  5 = 10-12 hours  6 = 13-15 hours  7 = almost the entire day  8 = do not know/do not want to answer |
| pa_sport_p | Do you exercise or do sports regularly (at least once a week)? | 1 = yes  2 = no  3 = do not know/do not want to answer |
| mobile_wd_p | How much time do you spend on the following during a normal weekday, from the time you wake up until you go to bed?  On the smartphone and/or tablet | 1 = no time  2 = 15 min or more  3 = 30 min  4 = 1 hour  5 = 2 hours  6 = 3 hours  7 = 4 hours  8 = 5 hours  9 = 6 hours or more |
| tv_wd_p | How much time do you spend on the following during a normal weekday, from the time you wake up until you go to bed?  Watch TV (including Netflix and Youtube) | 1 = no time  2 = 15 min or more  3 = 30 min  4 = 1 hour  5 = 2 hours  6 = 3 hours  7 = 4 hours  8 = 5 hours  9 = 6 hours or more |
| game_wd_p | How much time do you spend on the following during a normal weekday, from the time you wake up until you go to bed?  Playing computer or video games | 1 = no time  2 = 15 min or more  3 = 30 min  4 = 1 hour  5 = 2 hours  6 = 3 hours  7 = 4 hours  8 = 5 hours  9 = 6 hours or more |
| music_wd_p | How much time do you spend on the following during a normal weekday, from the time you wake up until you go to bed?  Sitting listening to music (e.g. Spotify/CD) | 1 = no time  2 = 15 min or more  3 = 30 min  4 = 1 hour  5 = 2 hours  6 = 3 hours  7 = 4 hours  8 = 5 hours  9 = 6 hours or more |
| talk_wd_p | How much time do you spend on the following during a normal weekday, from the time you wake up until you go to bed?  Sitting and talking on the phone | 1 = no time  2 = 15 min or more  3 = 30 min  4 = 1 hour  5 = 2 hours  6 = 3 hours  7 = 4 hours  8 = 5 hours  9 = 6 hours or more |
| paper_wd_p | How much time do you spend on the following during a normal weekday, from the time you wake up until you go to bed?  Doing paper work or computer work (office work, emails, paying bills etc.) | 1 = no time  2 = 15 min or more  3 = 30 min  4 = 1 hour  5 = 2 hours  6 = 3 hours  7 = 4 hours  8 = 5 hours  9 = 6 hours or more |
| book_wd_p | How much time do you spend on the following during a normal weekday, from the time you wake up until you go to bed?  Sitting reading a book or magazine | 1 = no time  2 = 15 min or more  3 = 30 min  4 = 1 hour  5 = 2 hours  6 = 3 hours  7 = 4 hours  8 = 5 hours  9 = 6 hours or more |
| instrument_wd_p | How much time do you spend on the following during a normal weekday, from the time you wake up until you go to bed?  Playing a musical instrument | 1 = no time  2 = 15 min or more  3 = 30 min  4 = 1 hour  5 = 2 hours  6 = 3 hours  7 = 4 hours  8 = 5 hours  9 = 6 hours or more |
| art_wd_p | How much time do you spend on the following during a normal weekday, from the time you wake up until you go to bed?  Doing artwork or crafts (e.g. painting, knitting etc.) | 1 = no time  2 = 15 min or more  3 = 30 min  4 = 1 hour  5 = 2 hours  6 = 3 hours  7 = 4 hours  8 = 5 hours  9 = 6 hours or more |
| car_wd_p | How much time do you spend on the following during a normal weekday, from the time you wake up until you go to bed?  Sitting and driving in a car, bus, train or other motorized vehicle | 1 = no time  2 = 15 min or more  3 = 30 min  4 = 1 hour  5 = 2 hours  6 = 3 hours  7 = 4 hours  8 = 5 hours  9 = 6 hours or more |
| mobile_we_p | How much time do you spend on the following during a normal weekend, from the time you wake up until you go to bed?  On the smartphone and/or tablet | 1 = no time  2 = 15 min or more  3 = 30 min  4 = 1 hour  5 = 2 hours  6 = 3 hours  7 = 4 hours  8 = 5 hours  9 = 6 hours or more |
| tv_we_p | How much time do you spend on the following during a normal weekend, from the time you wake up until you go to bed?  Watch TV (including Netflix and Youtube) | 1 = no time  2 = 15 min or more  3 = 30 min  4 = 1 hour  5 = 2 hours  6 = 3 hours  7 = 4 hours  8 = 5 hours  9 = 6 hours or more |
| game_we_p | How much time do you spend on the following during a normal weekend, from the time you wake up until you go to bed?  Playing computer or video games | 1 = no time  2 = 15 min or more  3 = 30 min  4 = 1 hour  5 = 2 hours  6 = 3 hours  7 = 4 hours  8 = 5 hours  9 = 6 hours or more |
| music_we_p | How much time do you spend on the following during a normal weekend, from the time you wake up until you go to bed?  Sitting listening to music (e.g. Spotify/CD) | 1 = no time  2 = 15 min or more  3 = 30 min  4 = 1 hour  5 = 2 hours  6 = 3 hours  7 = 4 hours  8 = 5 hours  9 = 6 hours or more |
| talk_we_p | How much time do you spend on the following during a normal weekend, from the time you wake up until you go to bed?  Sitting and talking on the phone | 1 = no time  2 = 15 min or more  3 = 30 min  4 = 1 hour  5 = 2 hours  6 = 3 hours  7 = 4 hours  8 = 5 hours  9 = 6 hours or more |
| paper_we_p | How much time do you spend on the following during a normal weekend, from the time you wake up until you go to bed?  Doing paper work or computer work (office work, emails, paying bills etc.) | 1 = no time  2 = 15 min or more  3 = 30 min  4 = 1 hour  5 = 2 hours  6 = 3 hours  7 = 4 hours  8 = 5 hours  9 = 6 hours or more |
| book_we_p | How much time do you spend on the following during a normal weekend, from the time you wake up until you go to bed?  Sitting reading a book or magazine | 1 = no time  2 = 15 min or more  3 = 30 min  4 = 1 hour  5 = 2 hours  6 = 3 hours  7 = 4 hours  8 = 5 hours  9 = 6 hours or more |
| instrument_we_p | How much time do you spend on the following during a normal weekend, from the time you wake up until you go to bed?  Playing a musical instrument | 1 = no time  2 = 15 min or more  3 = 30 min  4 = 1 hour  5 = 2 hours  6 = 3 hours  7 = 4 hours  8 = 5 hours  9 = 6 hours or more |
| art_we_p | How much time do you spend on the following during a normal weekend, from the time you wake up until you go to bed?  Doing artwork or crafts (e.g. painting, knitting etc.) | 1 = no time  2 = 15 min or more  3 = 30 min  4 = 1 hour  5 = 2 hours  6 = 3 hours  7 = 4 hours  8 = 5 hours  9 = 6 hours or more |
| car_we_p | How much time do you spend on the following during a normal weekend, from the time you wake up until you go to bed?  Sitting and driving in a car, bus, train or other motorized vehicle | 1 = no time  2 = 15 min or more  3 = 30 min  4 = 1 hour  5 = 2 hours  6 = 3 hours  7 = 4 hours  8 = 5 hours  9 = 6 hours or more |
| screen_child_p | Does your child have access to screens (TV, tablet, computer, mail etc.)? | 1 = yes  2 = no  3 = do not know/do not want to answer |
| screen_family_p | Do you as a family use screens during dinner (TV, tablet, computer, mobile, etc.)? | 1 = no, never  2 = yes, but only on weekends  3 = yes, 1-2 daily dinners/week  4 = yes, 3-4 daily dinners/week  5 = yes, 5-6 dinners/week  6 = yes, every dinner  7= do not know/do not want to answer |
| screen_bed_child_p | Does your child have the mobile phone in his/her bedroom during the night when he/she sleeps? | 1 = no, never  2 = yes, but only on weekends  3 = yes, 1-2 weekday evenings/week  4 = yes, 3-4 weekday evenings/week  5 = yes, 5-6 evenings/week  6 = yes, every evening  7= do not know/do not want to answer |
| screen_sleep_child_p | Does your child use any type of screen to fall asleep (mobile, TV, tablet, computer, etc.)? | 1 = no, never  2 = yes, but only on weekends  3 = yes, 1-2 times/week  4 = yes, 3-4 times/week  5 = yes, 5-6 times/week  6 = yes, every evening  7 = do not know/do not want to answer |
| wakeup_wd_child_p | When does your child usually get up from bed on a normal weekday? | 1 = earlier than 06:00  2 = 06:00-06:30  3 = 06:30-07:00  4 = 07:00-07:30  5 = 07:30-08:00  6 = 08:00-08:30  7 = 08:30-09:00  8 = later than 09:00  9 = do not know/do not want to answer |
| bedtime_wd_child_p | When does your child usually go to sleep a normal weekday? | 1 = earlier than 21:00  2 = 21:00-21:30  3 = 21:20-22:00  4 = 22:00-22:30  5 = 22:30-23:00  6 = later than 23:00  7 = do not know/do not want to answer |
| wakeup_we_child_p | When does your child usually get up from bed on the weekend? | 1 = earlier than 07:00  2 = 07:00-07:30  3 = 07:30-08:00  4 = 08:00-08:30  5 = 08:30-09:00  6 = 09:00-09:30  7 = 09:30-10:00  8 = 10:00-10:30  9 = later than 10:30  10 = do not know/do not want to answer |
| bedtime_we_child_p | When does your child usually go to sleep on the weekend? | 1 = earlier than 21:00  2 = 21:00-21:30  3 = 21:20-22:00  4 = 22:00-22:30  5 = 22:30-23:00  6 = 23:00-23:30  7 = later than 23:30  8 = do not know/do not want to answer |
| act_org_child_p | Does your child go to any organized sports or sport activity outside of school? | 1 = no  2 = yes, once a week  3 = yes, 1-2 times/week  4 = yes, 2-3 times/week  5 = yes, 3-4 times/week  6 = yes, 4-5 times/week  7 = yes, 5-6 times/week  8 = yes, every day  9 = do not know/do not want to answer |
| pa_evening_child_p | How physically active is your child after school or in the evening a normal weekday (walking, dancing, gymnastics, sports, other similar activity)? | 1 = not active at all, most sedentary  2 = less than 30 minutes  3 = 30-60 minutes  4 = 1-2 hours  5 = 2-3 hours  6 = more than 3 hours  7 = do not know/do not want to answer |
| pa_we_child_p | How physically active is your child usually on the weekend (walking, dancing, gymnastics, sports, other similar activity)? | 1 = not active at all, most sedentary  2 = less than 30 minutes  3 = 30-60 minutes  4 = 1-2 hours  5 = 2-3 hours  6 = more than 3 hours  7 = do not know/do not want to answer |
